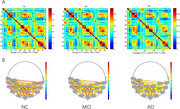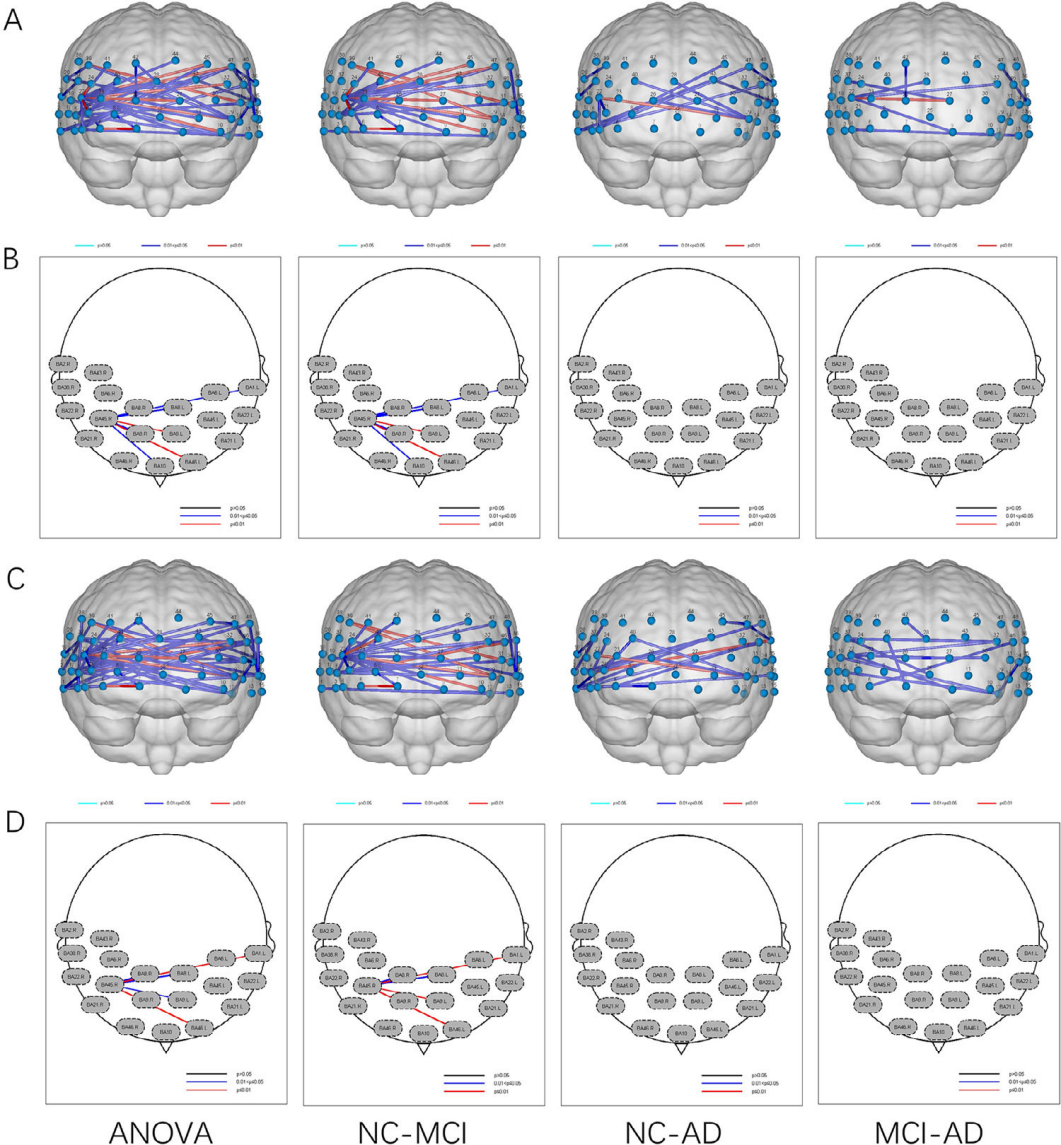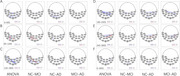# Correlation of prefrontal‐based resting state functional connectivity with the degree of cognitive impairment in Alzheimer's disease: a functional near‐infrared spectroscopy study

**DOI:** 10.1002/alz70856_101537

**Published:** 2025-12-25

**Authors:** Yang Lü, Ming Chen, Wenbo Zhang, Weihua Yu

**Affiliations:** ^1^ The First Affiliated Hospital of Chongqing Medical University, Chongqing, Chongqing, China; ^2^ Chongqing Medical University, Chongqing, Chongqing, China

## Abstract

**Background:**

This study aims to investigate the differences in neural network connectivity within the prefrontal cortex (PFC) among elderly individuals with normal cognition (NC), mild cognitive impairment (MCI), and Alzheimer's disease (AD) using functional near‐infrared spectroscopy (fNIRS), and to explore correlations with specific cognitive sub‐domains.

**Method:**

Resting‐state fNIRS measurements were conducted using a 48‐channel setup in 328 participants, including 78 participants with NC, 121 with MCI, and 129 with AD. All participants underwent a comprehensive neuropsychological battery. Functional connectivity (FC) between Brodmann area (BA) was analyzed. Multivariate logistic regression and linear regression were used to explore the associations between cognitive domain and FC of specific BAs.

**Result:**

In the resting state, there was no significant difference in the mean HbO_2_ in the PFC between the NC and MCI groups. However, AD patients exhibited higher mean HbO_2_ levels in the PFC. Significant differences in FC strength were observed between the NC, MCI, and AD groups in several BA pairs, including BA46.L‐BA45.R, BA9.L‐BA1.L, and BA21.R‐BA6.R. The most pronounced FC strength differences between the NC and MCI groups occurred at the 2^nd^‐minute mark, with BA45.R identified as a key node, while differences between the MCI and AD groups peaked at the 5^th^‐minute mark, with BA1.L as the key node. Additionally, the NC and MCI groups displayed significant FC strength differences in the prefrontal network during the first 2 minutes and first 3 minutes, again with BA45.R being central. Pearson's correlation analysis showed that FC strength between BA46.L‐BA45.R and BA9.L‐BA1.L was positively correlated with Mini‐Mental State Examination scores, while FC strength between BA9.L‐BA1.L was negatively correlated with Neuropsychiatric Inventory scores.

**Conclusion:**

Different resting‐state prefrontal FC strength were observed at different cognitive levels, and FC strength in the left dorsolateral PFC, where BA46.L and BA9.L are located, emerged as a key region for cortical dysfunction in cognitive impairment. Significant variations in prefrontal FC strength were identified across different time windows of the resting state, particularly highlighting the importance of BA45.R in distinguishing MCI from NC.